# Genome-Wide Association Study Reveals Novel Loci Associated with Body Conformation Traits in Qinchuan Cattle

**DOI:** 10.3390/ani13233628

**Published:** 2023-11-23

**Authors:** Hengwei Yu, Shengchen Yu, Juntao Guo, Gong Cheng, Chugang Mei, Linsen Zan

**Affiliations:** 1 College of Animal Science and Technology, Northwest A&F University, Xianyang 712100, China; yuhengwei@nwafu.edu.cn (H.Y.); yusc2023@nwafu.edu.cn (S.Y.); juntao@nwafu.edu.cn (J.G.); chenggong@nwafu.edu.cn (G.C.); 2 College of Grassland Agriculture, Northwest A&F University, Xianyang 712100, China; meichugang@163.com; 3 National Beef Cattle Improvement Center, Xianyang 712100, China

**Keywords:** GWAS, Qinchuan cattle, body conformation traits

## Abstract

**Simple Summary:**

The meat production of beef cattle is a major economic factor that is closely linked to body conformation traits. Qinchuan cattle are a local breed in China with a unique flavor. However, meat production needs improvement compared to commercial breeds. In this study, we selected 254 individuals, measured 14 phenotypic data points, and used genome-wide association analysis to identify loci and genes closely related to these traits. In total, we identified 250 suggestive loci and 37 candidate genes. These genetic markers will aid in the exploration of traits for Qinchuan cattle and provide a reference for breeding.

**Abstract:**

A genome-wide association study (GWAS) is an effective tool for identifying the dominant genes of complex economic traits in livestock by statistical analysis of genotype data and measured phenotype data. In this study, we rigorously measured 14 body conformation traits in 254 Qinchuan cattle, comprising body weight (BW), body height (BOH), back height (BAH), buttock height (BUH), chest depth (CD), chest width (CW), hip cross height (HCH), body length (BL), hip width (HW), rump length (RL), pin bone width (PBW), chest girth (CG), abdomen circumference (AG), and calf circumference (CC). After quality control, 281,889 SNPs were generated for GWAS with different traits. A total of 250 suggestive SNPs (*p* < 3.54 × 10^−6^) were screened and 37 candidate genes were annotated. Furthermore, we performed a linkage disequilibrium analysis of SNP loci and considered published studies, identifying the eight genes (*ADAMTS17*, *ALDH1A3*, *CHSY1*, *MAGEL2*, *MEF2A*, *SYNM*, *CNTNAP5*, and *CTNNA3*) most likely to be involved in growth traits. This study provides new insights into the regulatory mechanisms of bovine body size development, which can be very useful in the development of management and breeding strategies.

## 1. Introduction

Cattle are widely farmed around the world for meat, milk, skin, and leather. For beef cattle, meat yield and quality are the main concerns [1,2]. Several studies have shown that meat production in beef cattle is strongly correlated with body conformation traits, including body weight, body height, body length, chest circumference and more, which can be used as a measure of beef cattle size [3,4]. Therefore, it is of great significance for the livestock industry to study the body characteristics of cattle. China is a leading country globally for beef cattle breeding, characterized by large numbers of stalls and abundant local germplasm resources. Thus far, there are 55 local cattle breeds in China, of which Qinchuan cattle is one of the representative local beef cattle resources. There are many studies on the genome analysis of Qinchuan cattle, including selection signal analysis [5] and genetic polymorphism [6,7]. It is helpful to analyze the genetic basis of bovine variation among individuals for breeding and improving the economic efficiency of domestic breeds.

Cattle body conformation traits are comprehensively and delicately regulated by multiple genes, and it is a considerable inconvenience to find the main effector genes; however, a genome-wide association study (GWAS) can be used for the genetic analysis of economic traits and breeding. A GWAS is a statistical analysis method that associates genome-wide genetic variations with phenotypic traits and selects genetic variations that are significantly related to phenotypic traits [8]. By integrating phenotypic and genotypic data, the mutation sites associated with traits are selected and candidate genes are identified by GWAS, thus greatly contributing to the analysis of complex agricultural traits in farm animals. For example, a GWAS was performed on 18,274 animals across 10 US beef cattle breeds to study body weights [9]. A GWAS was also conducted on 1217 Simmental beef cattle to investigate the variation in abdominal size, body height, body length, hip height, and cannon bone size during different stages of growth with the use of LONG-GWAS, single-trait GWAS, and multi-trait GWAS, which revealed 58 significant SNPs compatible with 21 genes correlated to body size [10]. Chen et al. conducted a GWAS of 15 body size traits using autosomal SNPs derived from whole-genome sequences of 31 Brahman cattle and 131 Yunling cattle and identified 20 significant loci, which implicated 18 candidate genes [11]. A large body of evidence has conclusively determined that complex phenotypes require large amounts of genomic data to analyze the genetic loci by GWAS.

However, comprehensive research on the body size traits of indigenous Chinese cattle is still lacking. Qinchuan cattle have a significantly larger body size index after many years of breeding compared to non-core groups [12], and they are an ideal model to investigate the genetic basis of complex traits. In this study, we measured 14 body conformation traits in 254 individuals for a comprehensive analysis of body type, comprising body weight (BW), body height (BOH), back height (BAH), buttock height (BUH), chest depth (CD), chest width (CW), hip cross height (HCH), body length (BL), hip width (HW), rump length (RL), pin bone width (PBW), chest girth (CG), abdomen circumference (AG), and calf circumference (CC). This study provides a new insight into the genetic variation in differences in body size among beef cattle individuals and the genetic improvement of domestic cattle.

## 2. Materials and Methods

### 2.1. Ethics Statement

Ethics approval for all animal experiments was granted by the Institutional Animal Care and Use Committee of Northwest A&F University following the recommendations of the Regulations for the Administration of Affairs Concerning Experimental Animals of China.

### 2.2. Animals

We collected blood samples and measured each body trait from 254 individuals, including the new strain of Qinchuan cattle (QNC, n = 215), unselected Qinchuan cattle (QCC, n = 20), and Zaosheng cattle (ZSC, n = 19), from the National Beef Cattle Improvement Center’s experiment farm (Yangling, China), the Genetic Resource Conservation of Qinchuan Cattle (Fufeng, China), and Longshang Tianyuan Agriculture and Animal Husbandry Co., Ltd. (Zhengning, China), respectively. These experimental animals were raised in the same environment. When selecting samples, close relationships between individuals were avoided. The cattle were placed in a hold frame or head lock and sterilized with an alcohol cotton ball. Then, blood samples were taken from the jugular vein, collected by negative pressure into EDTA-containing 5-milliliter gatherers, gently mixed in reverse several times, and, in the case of ice packs, transferred for long-term storage at −20 °C for DNA extraction.

### 2.3. Body Conformation Traits Assessment

The body conformation traits were assessed as follows. BW: weight on the same weighbridge (minimum scale 5 kg) before morning feeding; BOH: vertical height from the apex of the bun nail to the ground, measured by a measuring stick; BAH: vertical height of the last section of the thoracic spine to the ground, measured by means of a measuring stick; BUH: vertical height of the outer edge of the hip to the ground, measured by means of a measuring stick; CD: distance between the upper and lower chest of the back of the scapula, measured with a measuring stick; CW: distance between the shoulder blades measured by a measuring stick; HCH: the height of the ground vertically between the corners of the waist, measured by means of a measuring stick; BL: shoulder-to-hip distance, measured with a measuring stick; HW: the distance between the outer edges of the two waist corners, measured by a measuring stick; RL: the distance between the front edge of the waist corner and the back edge of the sciatica node, measured with a measuring stick; PBW: the distance between pin bones, measured by means of a measuring stick; CG: chest circumference measured by a tape measure along the back corner of the shoulder blade, the tightness of which was determined by sliding up and down the index and middle fingers; AG: a vertical line at the back edge of the waist was made and measured with a tape measure; CC: horizontal circumference was measured by a tape measure at 1/3 of the thinnest part of the left forearm tub.

### 2.4. Descriptive Statistics and Normality Testing of Phenotypic Data

In total, 14 phenotypic data points were statistically analyzed for number, mean, standard deviation, minimum, maximum, and coefficient of variation using Excel. If the absolute value of the difference between the measured value and the mean value was greater than 3 times the standard deviation, the measured value was considered an outlier and this data point was deleted. The frequency distribution histogram of each phenotype was drawn and tested for normality by R-4.2.1 software.

### 2.5. Genome Sequencing

Overall, genomes from 127 QNC underwent mutation detection using a 600 K high-density chip designed by our lab, and genomes from 88 QNC, 20 QCC, and 19 ZSC underwent 10× genome resequencing by Xinjiang Compass Agritechnology Co., Ltd. (Xinjiang, China). DNA extraction, detection, fragment purification, library construction, and whole genome sequencing using the BGI T7/G2000 technique with paired end reads of 150 bp from the samples were completed. Before subsequent analysis, we filtered the original data according to the following conditions: remove reads with adapters; when the N content in the sequencing read exceeds 10% of the base number of the read, remove the paired reads; when the number of low-quality (Q <= 5) bases contained exceeds 50% of the number of bases in the read, remove the paired reads.

### 2.6. Alignments and Variant Calling

Quality control of the clean data obtained from the company was performed using FastQC software version 0.12.0 with default parameters [13], and the mem2 parameters in the Burrows–Wheeler Aligner v0.7.17 (BWA) [14] aligned those clean reads to the cattle ARS-UCD1.2 reference genome. Reference genomes were indexed using the Genome analysis toolkit v4.0 (GATK) software [15], and gvcf files were generated using the “HaplotypeCaller” module. The gvcf files for multiple samples were merged according to chromosomes, and genotypes were generated using the “GenotypeGVCFs” module. SNPs were filtered using the “SelectVariants” module. Finally, hard filtering was performed using the “VariantFiltration” module with the parameters “QD < 2.0 || MQ < 40.0 || FS > 60.0 || SOR > 3.0 || MQRankSum < −12.5 || ReadPosRankSum < −8.0”. Genotype files obtained from the 600 K chip data and resequencing data were merged using BCFtools software v1.18 [16], then filled with beagle 5.4 [17] and filtered by DR2 > 0.9 & MAF > 0.05.

### 2.7. Group Stratification Testing

We used Plink v1.9 software [18] to convert vcf formatted files into a new PLINK binary file set and then constructed a genetic relationship matrix based on SNP genetic markers using genome-wide complex trait analysis (GCTA v1.94.1) software with the parameters “--make-grm --autosome-num 29” [19], and then performed PCA analysis with parameters “--grm test --pca 5”. The principal components PCA1 and PCA2 were demonstrated using the ggplot2 package of R software.

### 2.8. Genome-Wide Association Analysis and Visualization

We carried out the GWAS with a mixed linear model using the genome-wide efficient mixed-model association software package v0.94.1 (GEMMA) [20]. At the time of analysis, we incorporated field effects, the first and second principal components, months of age, and batch effects of sequencing in cattle into the model as covariates. Manhattan and QQ plots were plotted using the CMplot package [21] of R software. The threshold for the Bonferroni level of significance was 3.54 × 10^−6^ (1/281,889). LDBlockShow [22] was used to draw a linkage disequilibrium heatmap and meaningful statistics to locate candidate intervals with the parameters “-InVCF -InGWAS”.

### 2.9. Gene Annotation and Candidate Genes Search

In order to further analyze the function of SNPs, we used a perl script and identified significant SNPs in 100 kb upstream and downstream as candidate regions for screening genes based on the Ensembl database. Then, in order to determine the function of annotated genes, PubMed was used to search for relevant published papers with https://www.ncbi.nlm.nih.gov/pubmed (accessed on 1 October 2023).

## 3. Results

### 3.1. Phenotypes and Genotypes

**Figure 1:**
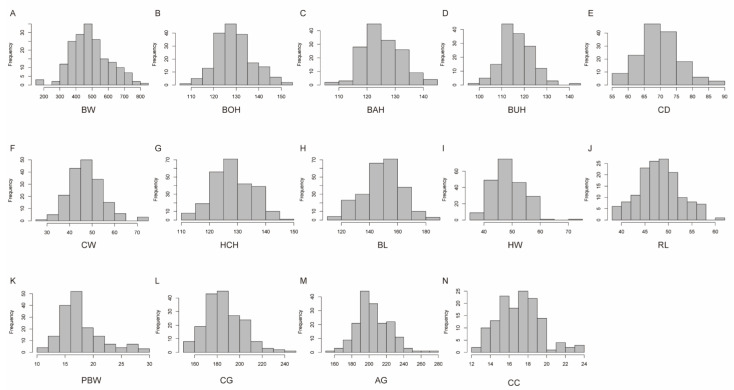
Histogram of phenotypic value frequency distribution of 14 body conformation traits. (**A**) BW, body weight. (**B**) BOH, body height. (**C**) BAH, back height. (**D**) BUH, buttock height. (**E**) CD, chest depth. (**F**) CW, chest width. (**G**) HCH, hip cross height. (**H**) BL, body length. (**I**) HW, hip width. (**J**) RL, rump length. (**K**) PBW, pin bone width. (**L**) CG, chest girth. (**M**) AG, abdomen circumference. (**N**) CC, calf circumference.

**Figure 2:**
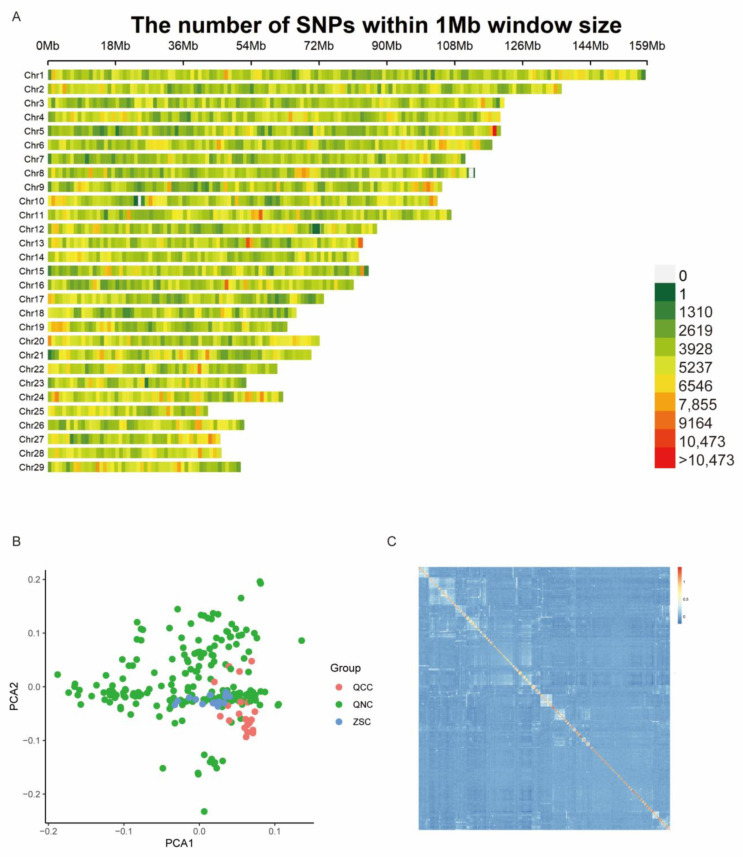
Distribution of SNPs in chromosomes and relationships between individuals. (**A**) The physical location of SNPs in the chromosome. (**B**) Population structure identified by principal components analysis. (**C**) Relationships between individuals.

A total of 254 cattle were measured for body conformation traits, and the descriptive statistics of the phenotypes are presented in Table 1. The mean and the standard deviation of BW (kg) and 13 other body conformation traits (BOH, BAH, BUH, CD, CW, HCH, BL, HW, RL, PBW, CG, AG, and CC) were 489.35 ± 118.66, 129.73 ± 8.26, 125.97 ± 6.99, 117.12 ± 6.83, 70.44 ± 6.57, 47.91 ± 7.41, 128.87 ± 6.97, 149.79 ± 13.43, 49.09 ± 5.73, 48.67 ± 4.50, 18.26 ± 3.86, 186.92 ± 16.85, 205.94 ± 19.21, and 17.58 ± 2.31, respectively. The coefficient of variation (CV) of body weight was the highest (24.25%), the CV of HCH was the lowest (5.41%), and that of the other traits ranged from 5.55% to 21.14%. The values of the phenotype data conformed to a Gaussian distribution for subsequent analysis (Figure 1). This study performed sequencing by a 600 K chip and 10× whole-genome sequencing (WGS) on 254 cattle, of which 127 were sequenced for a total of 4.3 TB raw data and 14 GB clean reads, and the average of Q20 was 98.21% (Supplementary Table S1). Combined microarray and WGS data yielded 11,316,457 SNPs, and the number of effective SNPs was 281,889 after quality control for GWAS. The SNPs were roughly evenly distributed across 29 chromosomes (Figure 2A).

### 3.2. Principal Components Analysis and Kinship Analysis

Samples underwent a principal components analysis (PCA) using GCTA software v1.94.1, and the top two components were visualized by R language, indicating no population stratification and no need for correction (Figure 2B). Kinship between samples was calculated using the Centralized_IBS method in the TASSEL software v5.0 and visualized using the pheatmap package in R. The relationships between individuals were considered to be relatively distant, so the affinity matrix did not need to be considered in the linear modified model (Figure 2C).

### 3.3. Genome-Wide Association Study for Body Conformation Traits

**Figure 3:**
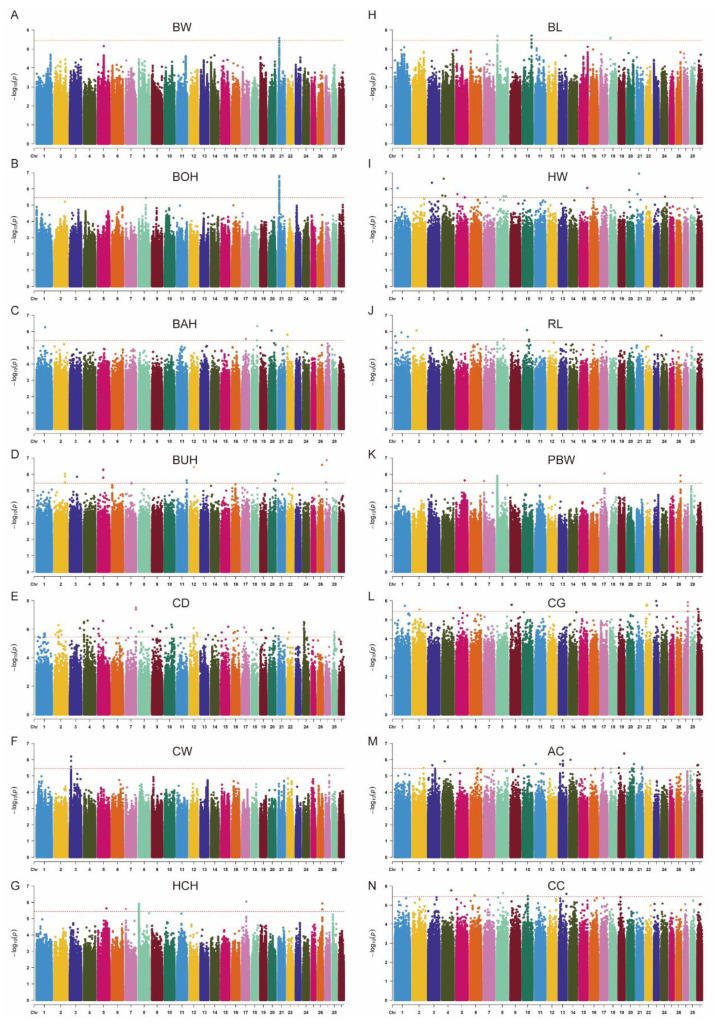
Manhattan plot of association results from genome-wide association analysis. (**A**) BW, body weight. (**B**) BOH, body height. (**C**) BAH, back height. (**D**) BUH, buttock height. (**E**) CD, chest depth. (**F**) CW, chest width. (**G**) HCH, hip cross height. (**H**) BL, body length. (**I**) HW, hip width. (**J**) RL, rump length. (**K**) PBW, pin bone width. (**L**) CG, chest girth. (**M**) AG, abdomen circumference. (**N**) CC, calf circumference. Y axis shows −log10 (*p*-value) of the association result for each SNP. The horizontal dashed line is the threshold for the Bonferroni level of significance (*p* < 3.54 × 10^−6^).

The Manhattan plot of association results from the genome-wide association analysis of each trait is shown in Figure 3. A total of 250 significant SNPs (*p* < 3.54 × 10^−6^) were screened for genome-wide association analysis of 14 body conformation traits in 254 cattle and annotated to 37 candidate genes (Supplementary Table S2 and Table 2). For BW, one SNP was found on chromosome 21, an unannotated gene (Figure 3A). Regarding BOH, 27 SNPs were found on chromosome 21, annotated to 29 genes including *ASB7*, *IGF1R*, *MEF2A*, and more (Figure 3B). For BAH, chromosomes 1, 17, 18, 20, and 22 showed one SNP each with no genes annotated (Figure 3C). As for BUH, 16 SNPs were found on ten different chromosomes and candidate genes were not annotated (Figure 3D). For CD, a total of 82 SNPs were annotated, with many SNPs concentrated on chromosome 24 (22 SNPs), and all SNPs were annotated to seven candidate genes: *ARHGAP26*, *CNTNA5*, *CTNNA3*, *FBXL17*, *GPHN*, *SEMA3Eh*, and *THSD7B* (Figure 3E). For CW, all ten significant SNPs were on chromosome 3 (Figure 3F). For HCH, 19 SNPs were distributed on five chromosomes, with the largest number on chromosome 8 (14 SNPs), but no genes were annotated (Figure 3G). Regarding BL, 10 SNPs were distributed on chromosomes 8, 10, and 18, and no genes were annotated (Figure 3H). For HW, 15 SNPs were distributed on nine chromosomes without gene annotation (Figure 3I). As for RL, nine SNPs were distributed on five chromosomes, annotated to one gene (*FRMD6*) (Figure 3J). Regarding PBW, 19 SNPs were distributed on five chromosomes, of which 14 SNPs were on chromosome 8, with unannotated genes (Figure 3K). For CG, there were 12 significant SNPs, annotated to one gene (*UBE2E2*) (Figure 3L). Eighteen and seven SNPs associated with AC and CC, respectively, were not annotated with candidate genes (Figure 3M,N). Overall, 66 SNPs, focused on chromosomes 3, 8, 21, and 24, deserve significant attention.

### 3.4. Linkage Disequilibrium Analysis of Key Candidate Area

**Figure 4:**
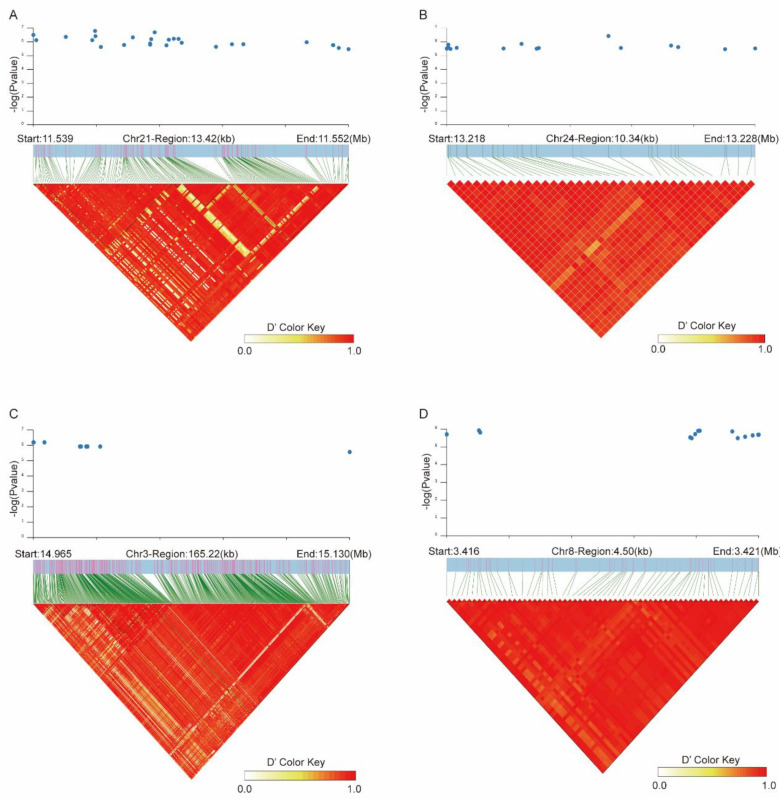
Haplotype block at linkage disequilibrium (LD) based on four candidate variations. (**A**) 21:11,538,587–11,552,009 on chr21 for BOH. (**B**) 24:13,218,042–13,228,386 on chr24 for CD. (**C**) 3:14,964,795–15,130,020 on chr 3 for CW. (**D**) 8:3,416,418–3,420,913 on chr8 for HCH. The small blue dots in the diagrams above the haplotype block indicate the genome-wide suggestive significant SNPs.

Using the GWAS for each trait, we found that 27 SNPs closely associated with BOH were concentrated on chromosome 21 (21:11,538,587–11,552,009), 15 SNPs strongly associated with CD were concentrated on chromosome 24 (24:13,218,042–13,228,386), 10 SNPs strongly associated with CW were concentrated on chromosome 3 (3:14,964,795–15,130,020), and 14 SNPs strongly associated with HCH were concentrated on chromosome 8 (8:3,416,418–3,420,913). The physical locations of these SNPs are very close together, and in order to explore the interlocking relationships between them, a linkage disequilibrium analysis was performed. The distribution of haplotypes is shown in Figure 4. For BOH, 187 SNPs on chromosome 21 formed three large haplotype blocks in the 13.42 kb range. For CD, there were 37 SNPs in the 10.34 kb range on chromosome 24, all of which were closely linked to form a haplotype block. As for CW, there were 553 SNPs that were in the 165.22 kb range on chromosome 3, forming two large haplotype blocks. For HCH, 63 SNPs in the 4.5 kb range were on chromosome 8, all closely linked to form a haplotype block.

## 4. Discussion

In general, animal weight and growth rates are considered profit drivers of animal production systems [23]. Quantitative traits such as the body weight and body size of beef cattle are important phenotypic data, which are vital to study the appearance and production performance of breeds and ultimately play a crucial role in breed selection and improvement. Body length and chest circumference had the greatest impact on BW, with phenotypic correlation coefficients of 0.975 and 0.962 [4], respectively.

Biological breeding can effectively increase the amount of meat production and improve the quality of meat per beef cattle, on the one hand saving feedstock and controlling breeding cost, and, on the other hand, improving the economic value of high-quality meat. However, the quantitative traits of beef cattle are co-regulated by multiple genes, which are harder to find. GWAS provides a good example of screening for dominant genes associated with quantitative traits in livestock and poultry, such as pigs [24,25], chickens [26,27,28], ducks [29], sheep [30,31], beef cattle [32,33], and cows [34,35,36]. Moreover, GWASs of beef cattle mainly assess growth traits [37], reproductive traits [38,39], and meat quality traits [40,41]. In this study, a number of prominent SNPs and candidate genes were detected among 14 body conformation traits.

We conducted a literature search of 37 selected candidate genes and identified several important genes that may be associated with quantitative traits in beef cattle. It has been firmly established that *ADAMTS17* is a pathogenic gene for Weill–Marchesani syndrome (WMS) and Weill–Marchesani-like syndrome, often characterized by lens heterotopia and small stature [42], and has been shown to regulate the function of profibrin microfibers. In addition, *ADAMTS17* is implicated in skeletal formation through modulation of the BMP-Smad1/5/8 pathway, possibly through inhibition of fibrillin-2 incorporation into microfibrils [43]. Two dairy cattle populations (Holstein Friesian and Jersey) were selected to discover CNVs using the Illumina BovineHD Genotyping BeadChip, and the population differentiation index revealed that *ADAMTS17* hints at adaptive evolution [44]. Likewise, CRISPR/Cas9 targeting and gene-specific knockdowns showed that *ALDH1A3* is a key enzyme involved in the production of retinoic acid required for head formation [45]. Phylogenetic studies supported a hypothesis for *ALDH1A2* as a probable primordial gene that originated in invertebrate genomes and underwent a sequential gene duplication event to generate two additional genes, *ALDH1A1* and *ALDH1A3*, in the majority of vertebrate genomes [46]. GWASs have identified hundreds of loci associated with height. However, identifying causal mechanisms is challenging, especially because highly relevant tissues, such as growth plates, are difficult to study. In one study, epigenetic analysis of mouse femoral growth plates identified a candidate causal variation (rs9920291) overlapping with open chromatin regions in *CHSY1*, and targeting human chondrocytes with CRISPR/Cas9 technology demonstrated that this locus modulates *CHSY1* expression suggesting it as a key gene for human growth [47]. Genotyping of 1225 Simmental cattle was performed using an Illumina BovineHD BeadChip with 770,000 SNPs by GWAS, resulting in *CHSY1* being identified as a candidate gene for bone weight [48]. Furthermore, 136 goats with records of kidding were selected for GWAS using the Illumina Caprine 50 K bead chip, and the GWAS results indicated that *GABRA5* plays a role in reproductive processes, giving it potential for use in marker-assisted selection programs in Markhoz goats [49]. In Luzhong mutton sheep, a genome-wide comparative analysis was performed between two groups with different fecundity to reveal candidate genes based on a high-density SNP chip, and *GABRG3* was related to teat number [50]. Genotyping of the *IGF1R* gene in 1716 sheep from six breeds in New Zealand revealed an association between lifespan and variation in *IGF1R* [51]. In addition, *MAGEL2* is a patrilineal regulatory gene that may play an important role in muscle maturation, according to a study of piglets [52], and it is expressed in the developing musculoskeletal system [53]. *MEF2A* has been shown in several studies to play a significant role in myocardial muscle [54], skeletal muscle [55], and smooth muscle formation and function [56]. *MEF2A* regulates the MEG3-DIO3 miRNA mega cluster-targeted PP2A signaling in bovine skeletal myoblast differentiation [57] and sheep myoblast proliferation [58]. In a GWAS, *CNTNAP5* was speculated to be a candidate gene associated with growth and carcass traits including body weight and body fat deposition in four Beninese indigenous cattle breeds [59], as well as hip cross height in Brahman cattle [11]. *CTNNA3* was associated with development and growth by iHS and ROH detection based on genome-wide SNP markers in Youzhou dark goats [60] and in Hu sheep [61], as well as skeletal muscle during chicken embryonic development [62]. A large body of published studies may provide evidence for certain genes involved in the regulation of body conformation traits.

A haplotype is a combination of alleles at multiple loci that are shared across the same chromosome. The distribution of haplotype blocks throughout the genome can reflect the genetic structure and variation of a population [63]. The haplotype of each chromosome is unique in that it contains a complete set of genetic information and is an aspect of genomics research. Understanding the combination and inheritance of different loci on a single chromosome or in a specific region of a single chromosome can improve the accuracy of the analysis of complex traits [64]. For instance, Khanyile et al. identified genes associated with adaptability by constructing haplotype block structures in Southern African village chicken populations and annotating the longest haplotype blocks [63]. In another report, Salem et al. constructed haplotypic block structures in Holstein cow populations and identified genes associated with milk yield traits [65]. In this study, we found that the most significant SNP concentrations were on certain chromosomes, such as chromosomes 3, 8, 21, and 24. Remarkably, we constructed haplotypes by a linkage disequilibrium analysis in chromosome 21 (21:11,538,587–11,552,009) associated with BOH, chromosome 24 (24:13,218,042–13,228,386) related to CD, chromosome 3 (3:14,964,795–15,130,020) associated with CW, and chromosome 8 (8:3,416,418–3,420,913) related to HCH. For BOH, three large haplotype blocks were formed within 13.42 kb on chromosome 21. For CD, a haplotype block was formed within 10.34 kb on chromosome 24. In the case of CW, two large haplotype blocks were formed within 165.22 kb on chromosome 3. Finally, for HCH, a haplotype block was formed within 4.5 kb on chromosome 8. These findings contribute to explaining genetic variation in cattle body conformation traits.

Therefore, based on functional studies of these candidate genes, we identify *ADAMTS17*, *ALDH1A3*, *CHSY1*, *MAGEL2*, *MEF2A*, *SYNM*, *CNTNAP5*, and *CTNNA3* as promising candidates affecting growth traits in beef cattle.

## 5. Conclusions

This study shows that a GWAS can be a powerful tool for identifying novel loci and unraveling the genetic basis of complex economic traits. Furthermore, the important candidate genes and molecular markers of body conformation traits were selected from the genome to analyze the potential genetic mechanism of dominant traits and provide a scientific basis for breeding improvement of beef cattle. According to published studies, eight genes (*ADAMTS17*, *ALDH1A3*, *CHSY1*, *MAGEL2*, *MEF2A*, *SYNM*, *CNTNAP5*, and *CTNNA3*) can be considered potential candidates for body conformation traits, which could serve as important marker information for genome selection and contribute to the improvement of cattle breeding programs.

## Figures and Tables

**Table 1:** Descriptive statistics of phenotypes.

Trait	Number	Mean	SD	Min	Max	CV
BW, kg	180	489.35	118.66	159	836	24.25%
BOH, cm	184	129.73	8.26	107	155	6.37%
BAH, cm	150	125.97	6.99	107	144	5.55%
BUH, cm	146	117.12	6.83	99	144	5.84%
CD, cm	147	70.44	6.57	56	88	9.32%
CW, cm	178	47.91	7.41	28	72	15.48%
HCH, cm	246	128.87	6.97	113	146	5.41%
BL, cm	245	149.79	13.43	110	187	8.96%
HW, cm	210	49.09	5.73	35	72	11.68%
RL, cm	148	48.67	4.50	38	62	9.25%
PBW, cm	166	18.26	3.86	11	30	21.14%
CG, cm	180	186.92	16.85	150	245	9.02%
AG, cm	173	205.94	19.21	157	274	9.33%
CC, cm	137	17.58	2.31	12	24	13.13%

SD, standard deviation. Abbreviations: BW, body weight; BOH, body height; BAH, back height; BUH, buttock height; CD, chest depth; CW, chest width; HCH, hip cross height; BL, body length; HW, hip width; RL, rump length; PBW, pin bone width; CG, chest girth; AG, abdomen circumference; CC, calf circumference; CV, coefficient of variation (SD/Mean).

**Table 2:** Candidate genes for body conformation traits in Qinchuan cattle.

Body Trait	Candidate Gene	Chromosome	Start	End	Function
BW	-	-	-	-	-
BOH	*ADAMTS17*	21	6,331,524	6,739,118	Bone development; adaptive evolution
	*ALDH1A3*	21	5,629,152	5,670,937	Head formation
	*ARRDC4*	21	8,734,781	8,749,130	-
	*ASB7*	21	5,970,734	6,023,292	-
	*ATP10A*	21	2,758,172	2,940,408	-
	*CERS3*	21	6,079,335	6,238,773	-
	*CHSY1*	21	5,285,464	5,375,159	Human height; bone weight
	*GABRA5*	21	4,267,204	4,359,559	Reproductive processes
	*GABRB3*	21	3,866,347	4,146,441	-
	*GABRG3*	21	4,945,634	5,195,923	Teat number
	*IGF1R*	21	7,780,293	8,080,394	Longevity
	*LINS1*	21	6,023,636	6,056,491	-
	*LRRC28*	21	7,226,600	7,416,484	Sheep reproduction
	*LRRK1*	21	5,492,271	5,620,369	-
	*LYSMD4*	21	6,821,223	6,827,752	-
	*MAGEL2*	21	1,205,086	1,208,637	Muscle maturity
	*MEF2A*	21	6,860,316	7,041,397	Bovine skeletal myoblast differentiation;
	*MKRN3*	21	1,160,774	1,163,589	-
	*NDN*	21	1,252,109	1,253,718	-
	*NR2F2*	21	10,562,379	10,575,195	Pig litter size
	*PGPEP1L*	21	8,090,744	8,103,335	-
	*SELENOS*	21	5,244,997	5,254,196	-
	*SNORD115*	21	2,199,356	2,199,436	-
	*SNORD116*	21	2,028,520	2,028,611	-
	*SNRPN*	21	1,937,647	1,961,037	-
	*SYNM*	21	7,581,549	7,606,637	Skeletal muscle hypertrophy
	*TTC23*	21	7,418,429	7,576,775	Boar fertility
	*UBE3A*	21	2,303,028	2,366,406	Regulating proliferation and apoptosis
BAH	-	-	-	-	-
CD	*ARHGAP26*	7	53,810,968	54,280,637	-
	*CNTNAP5*	2	76,113,318	76,921,405	Growth and carcass traits; hip cross height
	*CTNNA3*	28	22,282,909	24,121,400	Chicken embryonic development; growth traits in sheep and goats
	*FBXL17*	7	106,989,100	107,509,243	-
	*GPHN*	10	78,563,456	79,097,795	-
	*SEMA3E*	4	36,966,817	37,237,011	-
	*THSD7B*	2	59,511,857	60,421,786	-
CW	-	-	-	-	-
HCH	-	-	-	-	-
BL	-	-	-	-	-
HW	-	-	-	-	-
RL	*FRMD6*	10	44,297,021	44,564,023	Pork quality traits and fat deposition
PBW	-	-	-	-	-
CG	*UBE2E2*	27	42,324,254	42,691,907	Feeding behavior; ectopic-fat traits
AC	-	-	-	-	-
CC	-	-	-	-	-

## Data Availability

Raw sequencing data are available from the Genome Sequence Archive of the National Genomics Data Center, China National Center for Bioinformation/Beijing Institute of Genomics, Chinese Academy of Sciences (*GSA: CRA011831*), publicly accessible at https://ngdc.cncb.ac.cn/gsa (accessed on 10 July 2023).

## Supplementary Materials

The following supporting information can be downloaded at the following: https://www.mdpi.com/article/10.3390/ani13233628/s1, Table S1: Statistics of WGS data after Quality Control. Table S2: Significant SNPs associated with body traits.
